# Structural Relationships Among Marketing Stimuli, Pleasure Emotion, Trust in Seller, and Impulsive Buying in Sports Livestreaming E-Commerce: Evidence from University Students in Eastern China

**DOI:** 10.3390/bs16071081

**Published:** 2026-07-01

**Authors:** Xiaochen Li, Sang-Back Nam

**Affiliations:** Department of Sport Science, Hanyang University ERICA, 55 Hanyangdaehak-ro, Sangnok-gu, Ansan 15588, Gyeonggi-do, Republic of Korea; lixiaochen02@hanyang.ac.kr

**Keywords:** sports livestreaming e-commerce, impulsive buying, trust in seller, pleasure emotion, consumer herding tendency, perceived scarcity, hedonic benefits of sales promotion tools

## Abstract

As livestreaming e-commerce rapidly expands, impulsive buying has become increasingly prominent in online consumption. However, although prior studies have examined impulsive buying in general e-commerce contexts, little attention has been paid to impulsive buying in sports livestreaming e-commerce. Drawing on the stimulus-organism-response framework and Dual-System Theory, this study examined the associations among consumer herding tendency, perceived scarcity, hedonic benefits of sales promotion tools, pleasure emotion, trust in seller, and impulsive buying. A cross-sectional self-report survey was conducted with 775 university students in eastern China with prior purchase experience in sports livestreaming e-commerce, and the model was analyzed using partial least squares structural equation modeling. The results showed that consumer herding tendency, perceived scarcity, and hedonic benefits of sales promotion tools were positively associated with pleasure emotion and trust in seller. Perceived scarcity showed the strongest path coefficient with trust in seller. Trust in seller and pleasure emotion were positively associated with impulsive buying, whereas self-control was negatively associated with impulsive buying. Specific indirect associations through trust in seller were generally stronger than those through pleasure emotion. The moderating effect of self-control was not significant, and the gender-based multi-group analysis revealed no significant differences between male and female consumers. Given the cross-sectional self-report design, these findings should be interpreted as associations rather than causal effects.

## 1. Introduction

Digital technologies and mobile commerce have reshaped how consumers obtain product information and make purchase decisions (Paul & Rosenbaum, 2020; Xu et al., 2020). Within this broader transformation, livestreaming e-commerce has emerged as an interactive form of digital commerce that integrates real-time communication, product demonstration, entertainment, and instant purchasing functions (Geng et al., 2020; Sarstedt et al., 2016). The growing convergence of e-commerce platforms, social media, and short-video applications has further positioned livestreaming commerce as a highly interactive shopping environment and provides an important context for examining impulsive buying.

Sports livestreaming e-commerce has become an increasingly important form of sports-related retailing, as sports brands, retailers, and related sellers use livestreaming to present and sell sports products and related merchandise (Geng et al., 2020). Compared with general livestreaming commerce, sports livestreaming e-commerce may involve shopping opportunities embedded within sport-related content, collective interaction, fan-oriented communication, and event-based consumption contexts. In this setting, social cues, scarcity cues, and promotional cues may acquire more context-specific meanings and may be closely associated with impulsive buying within a limited time frame (Parboteeah et al., 2009). However, existing research has not sufficiently explained how marketing-related cues and evaluations in sports livestreaming e-commerce are associated with impulsive buying through consumers’ affective and trust-based psychological states.

Impulsive buying is an important topic in consumer behavior research (Y. Sun & Bao, 2023). Existing studies suggest that impulsive buying is typically an unplanned purchase behavior closely associated with external cues and immediate emotional arousal, and its decision-making process differs from planned purchasing, which is based on clearly defined needs and rational evaluation (Stern, 1962). Compared with goal-oriented rational decision-making, impulsive buying is characterized by greater spontaneity, situational dependence, and emotional motivation (Iyer et al., 2020). Although previous studies have documented the relationship between livestreaming shopping and impulsive buying, limited attention has been given to understanding how marketing-related cues and evaluations in sports livestreaming e-commerce are associated with consumers’ impulsive buying through pleasure emotion and trust in seller.

To examine impulsive buying-related associations in sports livestreaming e-commerce, this study adopts the stimulus–organism–response (SOR) framework and integrates dual-system theory (DST) as a complementary perspective. The SOR framework provides a structure for examining how consumer-perceived marketing-related constructs are associated with consumers’ internal psychological states and behavioral responses, while DST helps distinguish affective and trust-based evaluative processes within the organism component. Specifically, this study focuses on three stimulus-side consumer-perceived marketing-related constructs, namely consumer herding tendency, perceived scarcity, and hedonic benefits of sales promotion tools, and examines their associations with impulsive buying through pleasure emotion and trust in seller. These constructs are measured through consumers’ self-reported tendencies, perceptions, and evaluations in the recalled sports livestreaming shopping context. Questionnaire data were collected from university students in eastern China with prior experience purchasing sports-related products through livestreaming e-commerce, and the proposed research model was tested using partial least squares structural equation modeling (PLS-SEM). By comparing the roles of pleasure emotion and trust in seller, this study provides insights into both affective and trust-based psychological pathways related to impulsive buying in sports livestreaming e-commerce and offers practical implications for responsible marketing strategies in livestreaming sports platforms and sports brands.

## 2. Literature Review

### 2.1. SOR Model

The SOR model originates from environmental psychology. Its central premise is that external environmental stimuli are associated with individuals’ behavioral responses through internal cognitive and emotional states (Mehrabian & Russell, 1974; Mummalaneni, 2005). The SOR model has been widely applied to studies of consumers’ online purchasing behavior and has been shown to effectively explain consumers’ emotional and behavioral response mechanisms in complex environments (Laato et al., 2020; Mehrabian & Russell, 1974). In online retail research, Mummalaneni (2005) validated the explanatory power of the SOR framework by examining the relationships among website characteristics, consumers’ emotional states, and online shopping behavior. In the context of online impulsive buying, Parboteeah et al. (2009) found that virtual cues were associated with consumers’ urge to buy impulsively through perceived usefulness and pleasure. Dawson and Kim (2010) further indicated that online cues such as time-limited offers, discounts, and promotional reminders were significantly and positively associated with impulsive buying, while Flight et al. (2012) emphasized the importance of a favorable shopping atmosphere in triggering impulsive purchases.

With the development of livestreaming e-commerce, the application of the SOR model in digital consumption contexts has been further extended. In the post-pandemic business environment, online settings have made consumers more prone to impulsive buying motivations and online settings have been associated with a greater likelihood of such behavior (Laato et al., 2020). Lin et al. (2022), in the context of livestreaming shopping, conceptualized demand, convenience, interactivity, and playfulness as stimulus variables, perceived enjoyment as the organism variable, and impulsive buying intention as the response variable, thereby showing how livestreaming environmental conditions are associated with impulsive buying intention through internal psychological states. Furthermore, X. Li et al. (2021a), in their study of social commerce, found that social interaction was associated with impulsive buying intention through physical presence, social presence, and flow experience. Xia et al. (2024) further categorized livestreaming stimuli into social cues and media cues, demonstrating that streamer interaction, peer interaction, vividness, and authenticity were jointly associated with impulsive buying through pleasure, arousal, and perceived uncertainty. Chung et al. (2025) also showed that information quality and platform social presence were positively associated with consumers’ flow state, which in turn was associated with impulsive buying behavior.

It can thus be observed that, in the context of livestreaming e-commerce, the organism component within the SOR model is no longer confined to a single dimension of pleasure emotion. Instead, it has progressively evolved into a multidimensional psychological construct encompassing affective perception, cognitive evaluation, and immersive experience, thereby providing a more comprehensive and contextually appropriate theoretical framework for explaining consumers’ impulsive buying behavior.

### 2.2. Dual-System Theory

Dual-System Theory posits that individual judgment and decision-making are not governed by a single psychological mechanism, but are instead shaped by two functionally distinct processing systems. System 1 is characterized by fast, automatic, intuitive, and impulse-driven processing, and tends to generate immediate responses to consumer-perceived marketing-related constructs. In contrast, System 2 operates through a slower, more rational, and deliberative analytical process, enabling individuals to evaluate and regulate impulsive tendencies (Frankish, 2010). Within this theoretical framework, consumer responses can be understood as the outcome of the joint operation of affective-intuitive and cognitive-evaluative processes across different decision-making contexts (Cui et al., 2022; Metcalfe & Mischel, 1999; Rustichini, 2008).

In marketing and information systems research, Dual-System Theory has been widely used to distinguish affective and cognitive pathways in explaining consumer responses (Frankish, 2010; X. Li et al., 2021b). Existing studies have shown that external cues in livestreaming contexts, such as key opinion leader characteristics and streamer linguistic features, were significantly associated with consumers’ emotional responses, cognitive judgments, purchase intention, and sales performance (He & Jin, 2024; Luo et al., 2025). This suggests that, in livestreaming shopping environments characterized by high interactivity, strong stimulation, and dense information flows, consumer decision-making may involve the joint operation of affective and cognitive processes.

Furthermore, Dual-System Theory can provide a more fine-grained explanation of the internal psychological processing mechanism at the “organism” stage of the SOR model (He & Jin, 2024; X. Li et al., 2021b). In consumer behavior research, consumer-perceived marketing-related constructs may be associated not only with affective responses, such as pleasure emotion and emotional arousal, but also with cognitive evaluations, such as trust, perceived uncertainty, and perceived value (Ahmad & Lilani, 2025; Huang et al., 2024; L. Li et al., 2024; X. Li et al., 2021b; Ma et al., 2024; C. Wang et al., 2025b; Xia et al., 2024; Xu et al., 2020; Y. Zhou & Huang, 2023). Therefore, integrating Dual-System Theory into the SOR framework helps explain how affective and cognitive organism states are related to consumers’ impulsive buying responses in livestreaming e-commerce.

Based on Dual-System Theory, this study conceptualizes pleasure emotion as an immediate affective response that may arise rapidly in response to marketing cues and consumer-perceived stimulus-related factors in sports livestreaming shopping. Trust in seller, by contrast, reflects consumers’ confidence in the seller’s reliability, expertise, and integrity. However, in livestreaming contexts, trust is not necessarily a slow or fully deliberative judgment. Given the real-time and interactive nature of livestreaming shopping, trust can also form quickly through cues such as seller professionalism, product presentation, social feedback, and transaction assurance. Trust in seller may be associated with lower perceived uncertainty and purchase risk, making immediate purchase decisions feel safer and more acceptable. Together, pleasure emotion and trust in seller represent affective and trust-based evaluative processes within the organism component, providing two psychological pathways for understanding impulsive buying in sports livestreaming e-commerce.

### 2.3. Impulse Buying in Sports Livestreaming E-Commerce

Livestreaming e-commerce refers to a form of electronic commerce in which product presentation, promotion, and transactions are conducted through livestreaming platforms (Xu et al., 2020). Prior research has examined livestreaming e-commerce from several perspectives, including streamer characteristics, platform functions, social interaction, service quality, and trust, thereby providing important insights into consumer responses in livestreaming shopping contexts (Guo et al., 2025; Ji et al., 2024; Lu et al., 2025; Pan et al., 2022).

Against this backdrop, impulsive buying has gradually become an important issue in livestreaming e-commerce research. Impulsive buying generally refers to an immediate purchase behavior made by consumers without prior planning or sufficient deliberation (Karbasivar & Yarahmadi, 2011). Existing studies indicate that impulsive buying often emerges during the shopping process and is jointly shaped by consumer-perceived marketing-related constructs and internal psychological states (Badgaiyan & Verma, 2015; Parboteeah et al., 2009; Xu et al., 2020). Positive emotions, such as pleasure and satisfaction, have also been widely discussed as important psychological states related to impulsive buying (Saad & Metawie, 2015; Souiden et al., 2019).

Compared with general livestreaming e-commerce, sports livestreaming e-commerce is characterized by greater contextual specificity (Y. Liu et al., 2025). When consumers enter such livestreaming environments, they may not be motivated solely by shopping purposes, but also by obtaining sports-related content, engaging with sports culture, or participating in interactions (H. Liu et al., 2025). Sports brand live streaming can be understood as a representative form of this context, integrating sports content presentation, consumer interaction, and product sales within the same livestreaming environment (H. Liu et al., 2025; Wongkitrungrueng & Assarut, 2020). Unlike general product-oriented live streaming, streamers in sports livestreaming e-commerce often simultaneously perform the functions of product demonstration, sports scenario construction, and brand image communication, which may make consumers more attentive to external cues during content participation (Chung et al., 2025; Lin et al., 2022; Xia et al., 2024).

Existing research has shown that, in sports livestreaming e-commerce contexts, content quality, parasocial interaction, and perceived ease of use were associated with impulsive buying through flow experience, and that this relationship may be stronger among consumers with higher fan identification (H. Liu et al., 2025). Similarly, Z. Li et al. (2025) investigated sporting goods e-commerce live streaming and found that scene features, including visual appeal, presence, and scene-product matching, were related to consumers’ flow experience and purchase intention. W. Wang and Oh (2025) found that social media livestreaming characteristics, such as interactivity, entertainment, visualization, and professionalization, were associated with attitudes, trust, and purchase intention among sports consumers.

Nevertheless, prior sports-related livestreaming studies have paid relatively limited attention to consumer-perceived marketing-related cues and evaluations. Prior literature suggests that these cues may be particularly salient due to interactive fan communities, visible peer purchasing, real-time engagement, and sports-specific promotions, such as event-based limited-time offers, livestream-exclusive benefits, and athlete- or team-branded merchandise. Trust in seller is also especially important in this context, as consumers often have concerns about product authenticity, quality, brand credibility, and after-sales reliability. Accordingly, this study examines how consumer-perceived marketing-related cues and evaluations are associated with impulsive buying in sports livestreaming e-commerce through pleasure emotion and trust in seller.

## 3. Research Model and Hypotheses

### 3.1. Consumer Herding Tendency, Trust in Seller, and Pleasure Emotion

Consumer herding tendency captures consumers’ inclination to rely on, follow, or imitate other consumers’ choices when making purchase decisions (Avery & Zemsky, 1998; Banerjee, 1992). Thus, it reflects a consumer-side decision tendency rather than a perception of product availability or purchase opportunity. Prior research suggests that decision uncertainty and the observability of others’ behaviors are important conditions for the emergence of herding behavior (H. Sun, 2013). In livestreaming e-commerce contexts, consumers are often unable to directly inspect products, while information asymmetry and difficulties in product evaluation are associated with greater decision uncertainty (C.-H. Lee & Chen, 2021; Schlosser et al., 2006). At the same time, indicators such as the number of online viewers, comment interactions, likes, and other consumers’ real-time purchase information make others’ behaviors highly visible within livestreaming rooms (X. Wang et al., 2019; Xu et al., 2020). M. Ali and Amir (2024) further reported that visible cues such as consumer ratings and sales volume were significantly associated with herding behavior, and that this relationship was stronger under higher perceived uncertainty. These findings support the relevance of herding tendency in explaining consumer responses in livestreaming e-commerce contexts.

Consumer herding tendency may also be positively associated with trust in seller. In livestreaming e-commerce, when consumers observe a large number of others making purchases, providing positive feedback, or continuously participating, such collective behaviors may serve as a form of social proof. Such social proof may be associated with lower perceived uncertainty in purchase decision-making and more favorable trust-related judgments regarding the livestreaming selling entity (Zhu, 2022). Prior studies have shown that reference group behavior, social interaction, and livestreaming cues were associated with consumer trust and subsequent purchase responses (Yin et al., 2025). Wongkitrungrueng and Assarut (2020) also indicated that various value cues in live streaming were indirectly associated with consumers’ subsequent behavior through different forms of trust, including seller trust. In the context of sports livestreaming e-commerce, social proof may be particularly salient because of the event atmosphere, community interaction, and sense of collective participation. Therefore, consumers’ herding tendency may be positively associated with trust in seller. Based on this reasoning, the following hypothesis is proposed:

**H1.**  *Consumer herding tendency is positively associated with trust in seller.*

From the affective pathway perspective, consumer herding tendency may also be positively associated with consumers’ positive emotional experiences. Real-time interaction, collective participation, and synchronized purchasing in livestreaming shopping may be associated with a stronger sense of involvement and positive emotions such as excitement and pleasure. Xia et al. (2024) reported a significant positive association between peer interaction and consumers’ levels of pleasure and arousal in livestreaming contexts. G. Li et al. (2022) further reported an indirect association between interaction quality and impulsive purchase intention through pleasure emotion and arousal in livestreaming shopping. These findings suggest that social participation and group interaction in livestreaming environments are associated with both consumers’ cognitive judgments and immediate emotional responses.

In the context of sports livestreaming e-commerce, this relationship may be particularly relevant because consumers are embedded in a sports-specific and interactive viewing environment (H. Liu et al., 2025). Pleasure emotion can be understood as an affective response that may arise in relation to social cues in livestreaming shopping environments (Xia et al., 2024). Therefore, consumers with stronger herding tendencies may report stronger positive emotional experiences. H. Liu et al. (2025) further found that interaction and platform characteristics in sports livestreaming e-commerce are associated with impulsive buying through flow experience, with fan identification strengthening this effect. This suggests that, in sports livestreaming contexts, emotional experiences associated with group participation and social cues may be particularly relevant (H. Liu et al., 2025; Xia et al., 2024). Consumers’ herding tendency reflects their reliance on and imitation of others’ behaviors in decision-making contexts (M. Ali & Amir, 2024; Banerjee, 1992). In livestreaming shopping, consumers with stronger herding tendencies may be more sensitive to social cues and collective participation and may therefore report stronger pleasure emotion. Based on this reasoning, the following hypothesis is proposed:

**H2.**  *Consumer herding tendency is positively associated with pleasure emotion.*

### 3.2. Perceived Scarcity, Trust in Seller, and Pleasure Emotion

Perceived scarcity refers to consumers’ subjective perception that product quantity, purchase opportunities, or promotional benefits are limited. When many consumers are buying or planning to buy a product, consumers may perceive that available inventory is being depleted and that the product may soon become unavailable. Therefore, such items capture demand-induced scarcity rather than consumers’ tendency to follow others’ choices. Within the SOR model, scarcity can be regarded as an external stimulus associated with consumers’ internal cognitive and emotional states and subsequent behavioral responses (Kamboj et al., 2018; Mehrabian & Russell, 1974). In livestreaming e-commerce, scarcity cues are frequently presented through time-limited offers, limited quantities, countdowns, and exclusive discounts, making them salient in consumers’ decision-making (R. Zhou, 2025). Prior studies suggest that external livestreaming cues may contribute to consumers’ trust-related evaluations and subsequent responses (Feng et al., 2024; Qu et al., 2023).

From the cognitive pathway perspective, perceived scarcity may also serve as an informational cue in consumers’ evaluation of the seller. In livestreaming commerce, consumers form judgments about sellers through cues available in the livestreaming environment (Wongkitrungrueng & Assarut, 2020; Zhang et al., 2022). Trust in seller refers to consumers’ overall evaluation of the credibility of the livestreaming selling entity, including the streamer’s credibility and the merchant’s ability to reliably fulfill the transaction, rather than trust in the product, platform, or livestreaming room itself. Prior studies suggest that various external cues in livestreaming are associated with consumers’ trust-related evaluations and subsequent responses (X. Li et al., 2024; Wongkitrungrueng & Assarut, 2020; Zhang et al., 2022). Therefore, in sports livestreaming e-commerce, scarcity cues such as limited-edition jerseys, event co-branded products, and live-stream-exclusive purchase quotas may be interpreted as signals of limited availability or time-sensitive purchase opportunities (Qu et al., 2023; Wu et al., 2012; R. Zhou, 2025). However, this positive interpretation may depend on whether scarcity cues are perceived as credible. When consumers recognize scarcity appeals as persuasive tactics, their persuasion knowledge may be activated, leading them to question the credibility of the message (Aguirre-Rodriguez, 2013). If such claims are perceived as deceptive or manipulative, they may be associated with lower trust in the seller (Darke & Ritchie, 2007). Nevertheless, in sports livestreaming e-commerce, scarcity cues are often tied to product features or event contexts and are therefore more likely to be viewed as legitimate signals of limited availability. Based on this reasoning, the following hypothesis is proposed:

**H3.**  *Perceived scarcity is positively associated with trust in seller.*

From the affective pathway perspective, perceived scarcity may also be associated with consumers’ emotional responses. Scarcity cues can be associated with a sense of urgency and competitive arousal, and scarcity-induced purchase decisions may be accompanied by anticipated emotions such as rejoicing (Duong et al., 2025; Qu et al., 2023). Prior research has reported indirect associations between scarcity promotions and purchase intention through emotional experience (Qu et al., 2023; R. Zhou, 2025). From the perspective of Dual-System Theory, pleasure emotion is more closely associated with rapid affective responses arising in response to external cues (Metcalfe & Mischel, 1999). Thus, when consumers perceive a product as scarce, they may be more likely to experience immediate affective responses. In sports livestreaming e-commerce, this affective pathway may be particularly relevant because content quality and para-social interactivity were positively associated with viewers’ flow experience, immersion, and enjoyment, while the relationship between flow experience and impulsive buying was stronger among consumers with higher fan identification (H. Liu et al., 2025). Time-limited offers, limited quantities, and event-related products may be associated with stronger feelings of excitement and pleasure. Accordingly, the following hypothesis is proposed:

**H4.**  *Perceived scarcity is positively associated with pleasure emotion.*

### 3.3. Hedonic Benefits of Sales Promotion Tools, Trust in Seller, and Pleasure Emotion

Hedonic benefits of sales promotion tools refer to consumers’ promotion-specific evaluations of the enjoyable, exploratory, and value-expressive benefits derived from promotional tools such as discounts, coupons, gifts, and livestream-exclusive offers in sports livestreaming e-commerce. Rather than capturing the objective presence or intensity of promotions, this construct reflects the hedonic value consumers perceive from such tools. Prior research distinguishes hedonic benefits, such as value expression, entertainment, and exploration, from utilitarian benefits related to savings, convenience, and economic efficiency (Chandon et al., 2000; Sinha & Verma, 2020). Accordingly, the items used in this study capture consumers’ pride, enjoyment, pleasure, and willingness to try new brands under sales promotion conditions.

From a trust-related evaluative perspective, consumers’ perceived hedonic benefits of sales promotion tools may be positively associated with trust in seller. In livestreaming shopping, consumers often rely on visible cues to evaluate the seller and the reliability of the transaction (Zhang et al., 2022). In sports livestreaming e-commerce, event-based promotions, fan-exclusive benefits, livestream-only offers, and time-limited incentives may function as salient cues within an interactive shopping environment. When these promotional tools are perceived as enjoyable, exploratory, and value-expressive, consumers may form more favorable evaluations of the transaction experience. Such evaluations may contribute to stronger trust-related judgments toward the seller.

However, excessive or ambiguous discounts may increase consumers’ perceived risk (J. E. Lee & Stoel, 2014) or lower their perceived product quality (J. E. Lee & Chen-Yu, 2018). Consumers may also question the seller’s intent when promotional tactics are perceived as overly strategic or misleading due to their persuasion knowledge (Hardesty et al., 2007). Thus, the positive association between hedonic benefits of sales promotion tools and trust in seller may depend on promotion transparency, seller credibility, product involvement, and consumers’ persuasion knowledge. In the context of sports livestreaming e-commerce, sales promotion tools may be perceived not only as economic incentives but also as sources of enjoyment, exploration, and value expression, and may therefore be evaluated in hedonic terms. Accordingly, the following hypothesis is proposed:

**H5.**  *Hedonic benefits of sales promotion tools are positively associated with trust in seller.*

From the affective pathway perspective, consumers’ perceived hedonic benefits of sales promotion tools may also be associated with pleasure emotion (Sinha & Verma, 2020). This argument is consistent with prior research suggesting that online shopping cues may shape consumers’ affective and cognitive responses during the shopping experience (Eroglu et al., 2001; Mehrabian & Russell, 1974). Although these two constructs are conceptually related, they are treated in this study as reflecting different aspects of consumer psychology. Hedonic benefits of sales promotion tools refer to consumers’ promotion-specific evaluations of enjoyment, exploration, and value expression derived from promotional tools (Chandon et al., 2000), whereas pleasure emotion refers to consumers’ broader positive affective state during the sports livestreaming shopping experience (Mehrabian & Russell, 1974). Prior research suggests that sales promotions can generate hedonic benefits, including entertainment, exploration, and value expression, in addition to utilitarian benefits such as monetary savings and convenience (Chandon et al., 2000; Sinha & Verma, 2020). Moreover, research on shopping value indicates that hedonic value reflects the experiential, enjoyable, and fun-oriented aspects of shopping and is related to important retail outcomes (Babin et al., 1994; Jones et al., 2006). Therefore, when consumers perceive livestream-exclusive offers, instant discounts, or time-limited incentives as enjoyable, exploratory, or value-expressive, these promotion-specific evaluations may correspond to stronger pleasure emotion in the livestreaming shopping process. In sports livestreaming e-commerce, such promotional tools may be especially salient when they are connected with sport-related products, event-based consumption, or fan-oriented promotional contexts. Accordingly, the following hypothesis is proposed:

**H6.**  *Hedonic benefits of sales promotion tools are positively associated with pleasure emotion.*

### 3.4. Trust in Seller, Pleasure Emotion, and Impulsive Buying

Trust is a multidimensional concept that can be divided into cognitive trust and affective trust (Aiken, 2006). Cognitive trust is based on judgments of others’ competence and reliability, reflecting individuals’ reasoning from behavioral information in a specific context (Tu et al., 2020). Affective trust, by contrast, is grounded in positive emotions generated by the other party’s perceived care and concern (S. Chen et al., 2021). Prior studies have found that both cognitive trust and affective trust are positively associated with consumers’ online purchase intentions (Hao et al., 2023). Livestreaming shopping combines virtuality with real-time interaction. Consumers usually cannot directly verify product quality as they would in offline shopping and therefore rely more heavily on trust to reduce transaction risk and decision uncertainty (X.-B. Wang & Gu, 2020). When consumers develop a high level of trust in the selling entity’s credibility, the accuracy of product information, and its ability to reliably fulfill the transaction, this trust may be associated with lower purchase hesitation and a smoother decision-making process, corresponding to a greater likelihood of impulsive buying (X. Li et al., 2024). Existing research has shown that trust in livestreaming contexts is significantly and positively associated with consumers’ purchase intentions and impulsive buying behavior (C. Wang et al., 2025a; Yang et al., 2025). In sports livestreaming e-commerce, sports equipment, event-related merchandise, and functional products are typically highly information-dependent. Consumers therefore need to rely more strongly on trust in seller to complete their purchase judgments. Accordingly, in sports livestreaming e-commerce contexts, higher trust in seller is more likely to be positively associated with consumers’ impulsive purchase decisions. Based on this reasoning, the following hypothesis is proposed:

**H7.**  *Trust in seller is positively associated with impulsive buying.*

### 3.5. Self-Control and Impulsive Buying

Self-control refers to individuals’ ability to regulate and constrain their own responses, including the capacity to inhibit impulses, resist temptation, and adjust behavior (Tangney et al., 2018). Prior research has shown that self-control failure is one of the key causes of impulsive buying (Baumeister, 2002). When individuals are unable to effectively restrain immediate desires, they are more likely to make unplanned and emotionally driven purchase decisions (Baumeister, 2002). In consumption contexts, impulsive buying typically reflects a conflict between the desire for immediate gratification and long-term rational goals, and self-control is often discussed as a factor related to lower impulsive buying tendencies (Baumeister, 2002; Nagar, 2016). Consumers with higher self-control are generally better able to restrain immediate purchase impulses and avoid unplanned purchasing decisions. In sports livestreaming e-commerce, where real-time interaction, social cues, and hedonic benefits of sales promotion tools may intensify purchase temptation, self-control is expected to be negatively associated with consumers’ tendency to engage in impulsive buying. Accordingly, the following hypothesis is proposed:

**H8.**  *Self-control is negatively associated with impulsive buying.*

Pleasure emotion is often associated with a stronger sense of immediate gratification and a greater likelihood of impulsive buying (Flight et al., 2012; Huang et al., 2024). However, consumers differ in the extent to which they translate pleasure emotion into actual purchase behavior. Consumers with higher levels of self-control are better able to suppress immediate purchase impulses associated with pleasure emotion. Conversely, when individuals’ emotional responses and purchase desires exceed their self-control capacity, they are more likely to make irrational and impulsive purchase decisions (M. Li et al., 2020; Loewenstein, 1996). In sports livestreaming e-commerce, where interaction and immersion are salient features of the viewing experience, pleasure-related responses may be especially prominent. Therefore, self-control may be relevant as a potential boundary condition in the relationship between pleasure emotion and impulsive buying. Based on this reasoning, this study argues that self-control negatively moderates the relationship between pleasure emotion and impulsive buying. Specifically, the higher the level of self-control, the weaker the positive relationship between pleasure emotion and impulsive buying; conversely, the lower the level of self-control, the more likely pleasure emotion is more strongly associated with impulsive buying. Accordingly, the following hypothesis is proposed:

**H9.**  *Self-control negatively moderates the relationship between pleasure emotion and impulsive buying.*

### 3.6. Research Framework

**Figure 1:**
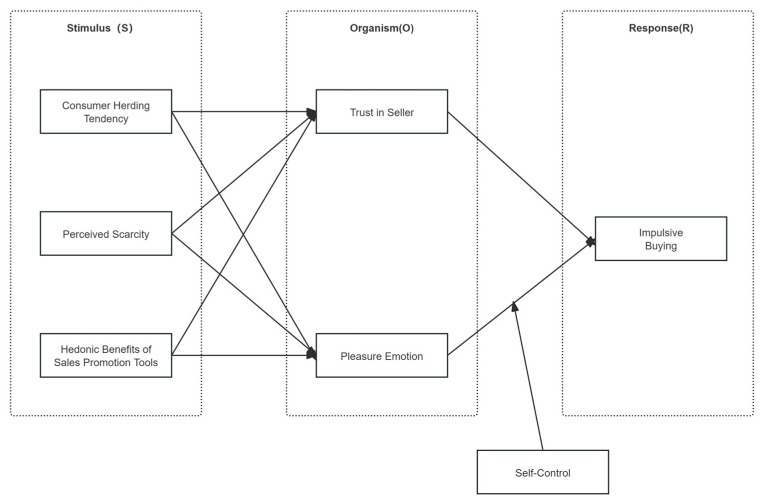
Research model.

Based on the SOR model, Dual-System Theory, and the hypotheses developed above, the research model of this study is proposed, as shown in Figure 1. Specifically, consumer herding tendency, perceived scarcity, and hedonic benefits of sales promotion tools are positioned within the stimulus (S) component as consumer-perceived marketing-related constructs, while trust in seller and pleasure emotion are positioned within the organism (O) component as psychological states. Pleasure emotion reflects consumers’ pleasure-related positive affective experience during sports livestreaming shopping, and impulsive buying is positioned as the response (R) variable. In addition, this study introduces self-control as a moderating variable and conducts gender-based multi-group analysis to examine whether the proposed associations differ across male and female respondents.

## 4. Method

### 4.1. Constructs and Measurement Items

The measurement items were adopted or adapted from prior studies. The constructs include three stimulus-side consumer-perceived marketing-related factors (X. Chen et al., 2025; Duong et al., 2025; Sinha & Verma, 2020; Wu et al., 2012), two organism factors (L. Li et al., 2024; Sullivan & Kim, 2018), one response factor (L. Li et al., 2024), and one moderating factor (S. Chen et al., 2022). The detailed measurement items and questionnaire sources are presented in Table 1 below.

### 4.2. Survey Design and Sampling Process

This study collected data through a questionnaire survey. The questionnaire consisted of three parts. The first part provided a brief introduction to sports livestreaming e-commerce, an explanation of impulsive buying, and a confidentiality statement. Respondents were instructed to complete the questionnaire based on their most recent sports livestreaming shopping experience. The second part collected respondents’ demographic and behavioral information, including gender, age, education level, monthly living expenses, prior purchase experience in sports livestreaming e-commerce, viewing frequency, and monthly expenditure on sports livestreaming e-commerce. The third part asked respondents to rate the measurement items listed in the table using a five-point Likert scale ranging from 1 = strongly disagree to 5 = strongly agree. All reverse-coded items were recoded before data analysis.

Since the survey was conducted in China, the original English questionnaire items were translated into Chinese using Brislin’s (1970) back-translation method. The original and back-translated versions were then compared to verify semantic consistency and ensure that no substantial differences in meaning existed. The initial questionnaire was submitted to professors in sport management for expert review and was revised based on their feedback. A pilot survey was then conducted using the revised version. A total of 130 questionnaires were distributed in the pilot survey. After excluding 12 invalid responses and 27 responses from participants without prior sports livestreaming e-commerce purchase experience, 91 valid responses remained. Based on these pilot data, the questionnaire was further refined to form the final version.

### 4.3. Data Collection and Sample Description

This study conducted an online survey to collect data for hypothesis testing. The research model was tested using data collected from university students through Wenjuanxing, a professional online survey platform in China, based on a purposive sampling approach. In this study, sports livestreaming e-commerce refers to livestreaming shopping contexts in which sports-related products are promoted and sold through real-time livestreaming platforms.

University students were selected as the sample for three main reasons. First, compared with other age groups, young consumers are more prone to impulsive emotions (Pechmann et al., 2005). Second, young people are major users of social media (Van Tran et al., 2023), and in the Internet era, they tend to rely more on social media communication than face-to-face communication (Punyanunt-Carter et al., 2018). Third, young consumers are generally more willing to accept new things and are more proficient in online shopping practices (Van Tran et al., 2023). Accordingly, the use of university students was appropriate for examining young consumers who are familiar with livestreaming platforms and online shopping. However, this sampling strategy was not intended to produce a nationally representative sample of all sports livestreaming e-commerce consumers.

Before completing the online questionnaire, respondents were informed of the research purpose, the voluntary nature of participation, the anonymity and confidentiality of their responses, and their right to withdraw from the survey at any time before submission. All respondents provided electronic informed consent before proceeding to the questionnaire. For respondents under the age of 18, informed consent from their parents or legal guardians was obtained with the assistance of school counselors before survey participation. No direct monetary or course-credit incentive was provided by the researchers.

For the purpose of this study, sports livestreaming e-commerce was understood as a livestreaming shopping context in which sports-related products or sports-related content was presented together with product promotion and purchasing opportunities. To reduce potential common method bias and improve response quality, several procedural remedies were incorporated into the questionnaire design and data collection process. Respondents were assured that there were no right or wrong answers, demographic and behavioral questions were separated from substantive measurement items, and only individuals with prior purchase experience in sports livestreaming e-commerce were eligible to participate. Before proceeding to the measurement items, respondents were asked to recall their most recent sports livestreaming shopping experience and answer the items with reference to that experience. Respondents from the same IP address were allowed to complete the questionnaire only once, and invalid responses were screened using completion time, identical response patterns, and logical consistency checks.

In total, 900 questionnaires were distributed. This study first excluded 17 invalid responses, including questionnaires completed in less than one minute, responses with identical answers throughout, and responses containing logical inconsistencies. It then excluded 108 responses from participants who answered “No” to the screening question. Ultimately, questionnaire data from 775 university students were included in the analysis, yielding a valid response rate of 86.11%.

The respondents were mainly located in eastern China, a relatively economically developed region with a high concentration of higher education institutions. A total of nine institutions were sampled, including two key universities, four regular universities, and three vocational colleges. The final sample of 775 valid responses was considered adequate for PLS-SEM because it substantially exceeded common minimum sample-size requirements. In the proposed model, the largest number of predictors pointing to a single endogenous construct was four, and the final sample size provided sufficient statistical power for estimating the structural model, including the structural paths and the interaction term.

As shown in Table 2, among the respondents, 449 were female and 326 were male, indicating a slightly higher proportion of female participants. Most respondents were aged between 19 and 22, and 82.7% held or were pursuing undergraduate or postgraduate qualifications. Since all respondents were university students, the sample generally reflects the typical characteristics of a student population. Specifically, 72.1% of respondents reported average monthly living expenses below RMB 3000, suggesting relatively limited disposable living expenses. Regarding the viewing frequency of sports livestreaming e-commerce, 83.6% of respondents watched such streams one to two times per week on average, indicating a relatively low overall viewing frequency. In terms of consumption level, 72.5% of respondents spent RMB 500 or less per month on livestreaming e-commerce, while 89% kept their monthly spending within RMB 1500. Overall, the sample is characterized by relatively low living expenses, low viewing frequency, and prudent consumption, which is consistent with the basic attributes of university students.

### 4.4. Data Analysis

Data screening, descriptive statistical analysis, and preliminary common method bias diagnostics, including Harman’s single-factor test, were conducted using IBM SPSS Statistics 29.0.1.0. The measurement model and structural model were assessed using SmartPLS 4.0. Specifically, reliability and validity were evaluated using Cronbach’s alpha, composite reliability, average variance extracted, the Fornell–Larcker criterion, and the heterotrait–monotrait ratio. The structural relationships, specific indirect effects, and the interaction effect related to self-control were tested using the bootstrapping procedure. In addition, model explanatory power and predictive relevance were assessed using R^2^, f^2^, and Q^2^predict. Finally, the MICOM procedure and gender-based multi-group analysis were conducted to examine measurement invariance and group differences.

## 5. Analysis and Results

### 5.1. Common Method Bias

Because all variables were collected from the same respondents using a cross-sectional self-report questionnaire, common method bias (CMB) was a potential concern. To reduce this risk, respondents were assured of anonymity and confidentiality, and data quality screening procedures were applied.

As a preliminary statistical assessment, Harman’s single-factor test was conducted by entering all measurement items into an unrotated principal component analysis in SPSS. The results showed that the first common factor explained 30.637% of the total variance, which was below the commonly used threshold of 40%. In addition, collinearity diagnostics were examined using the inner-model VIF values in SmartPLS. The results showed that all inner-model VIF values ranged from 1.004 to 1.294, below the recommended threshold of 3.3 (M. B. Ali et al., 2022; Sarstedt et al., 2016). Taken together, the procedural remedies and statistical diagnostics suggest that these preliminary diagnostics did not indicate severe common method bias. Nevertheless, these tests should be interpreted as preliminary diagnostics and do not by themselves eliminate the possibility of common method variance in a cross-sectional same-source survey design.

### 5.2. Test of the Measurement Model

This study employed Partial Least Squares Structural Equation Modeling (PLS-SEM) for data analysis. PLS-SEM is particularly suitable for prediction-oriented research, complex model analysis, and situations in which sample data may not satisfy the assumption of multivariate normality (M. B. Ali et al., 2022; Henseler et al., 2016). Accordingly, SPSS was first used to preprocess the sample data, and SmartPLS was then employed to assess both the measurement model and the structural model.

The results of the PLS-SEM measurement model assessment are presented in Table 3. The outer loadings of all latent variables ranged from 0.757 to 0.901, exceeding the recommended threshold of 0.70. This indicates that the observed variables adequately represent their corresponding latent constructs and that the measurement model demonstrates satisfactory indicator reliability. All latent constructs also showed strong internal consistency and convergent validity. Cronbach’s alpha values for all constructs exceeded 0.80, ranging from 0.809 to 0.899. The rho_a values ranged from 0.809 to 0.904, while composite reliability (rho_c) ranged from 0.875 to 0.929, all exceeding the recommended criteria. In addition, the average variance extracted (AVE) values ranged from 0.636 to 0.767, surpassing the critical threshold of 0.50 (Fornell & Larcker, 1981). This suggests that the latent variables adequately explain the variance in their corresponding measurement items. Overall, the measurement model demonstrates satisfactory internal consistency and convergent validity.

This study comprehensively assessed discriminant validity using the Fornell–Larcker criterion and the heterotrait–monotrait ratio (HTMT) (Fornell & Larcker, 1981). As shown in Table 4 and Table 5, the square root of the AVE for each latent variable was greater than the absolute values of its correlations with other latent variables, satisfying the Fornell–Larcker criterion (Fornell & Larcker, 1981). Meanwhile, the HTMT values among the constructs ranged from 0.020 to 0.571, all below the recommended threshold of 0.85, indicating satisfactory discriminant validity among the latent variables (Hair et al., 2019). Furthermore, the VIF values of the measurement items ranged from 1.544 to 2.835, all below 3.0. This suggests that there was no serious multicollinearity problem in the model and that the degree of overlap among the measurement indicators remained within an acceptable range (Dijkstra & Henseler, 2015). These results support empirical discriminant validity among the latent variables, while the content-based distinction between hedonic benefits of sales promotion tools and pleasure emotion is further addressed in the construct definitions, hypothesis development, and limitations section.

### 5.3. Structural Model Analysis

The results of the structural model assessment are presented in Table 6. The bootstrapping analysis indicated that most direct paths reached statistical significance (Soroya et al., 2021). From the paths between the stimulus variables and organism variables, the path coefficients from CHT to PE (β = 0.136, *t* = 3.489, *p* < 0.001) and TS (β = 0.138, *t* = 3.644, *p* < 0.001) were positive and statistically significant, supporting H1 and H2. The path coefficients from PS to PE (β = 0.233, *t* = 6.166, *p* < 0.001) and TS (β = 0.301, *t* = 8.812, *p* < 0.001) were also positive and statistically significant, supporting H3 and H4. Similarly, the path coefficients from HBSPT to PE (β = 0.215, *t* = 5.966, *p* < 0.001) and TS (β = 0.229, *t* = 6.976, *p* < 0.001) were positive and statistically significant, supporting H5 and H6.

From the paths between the organism variables and impulsive buying, PE (β = 0.275, *t* = 8.297, *p* < 0.001) and TS (β = 0.314, *t* = 9.084, *p* < 0.001) showed positive and statistically significant path coefficients with IB, supporting H7. The path coefficient from SC to IB was negative and statistically significant (β = −0.154, *t* = 4.819, *p* < 0.001), supporting H8. By contrast, the interaction path coefficient for SC × PE→IB was not statistically significant (β = −0.053, *t* = 1.629, *p* = 0.103). Therefore, H9 was not supported.

The explanatory power of the structural model was assessed using the coefficient of determination (R^2^), while effect sizes were evaluated using the f^2^ statistic. As shown in Table 7, the model explained 30.6%, 20.4%, and 27.0% of the variance in IB, PE, and TS, respectively, indicating that the model has a certain level of explanatory power for the endogenous variables. Overall, the f^2^ values of all paths fell within the small-effect range. Among them, the effect size of TS on IB was relatively higher (f^2^ = 0.118), followed by PS on TS (f^2^ = 0.096) and PE on IB (f^2^ = 0.092). By contrast, the effect size of SC × PE on IB was the smallest (f^2^ = 0.004), suggesting that its practical contribution was limited.

The predictive ability of the model was further assessed using the PLSpredict procedure in SmartPLS 4 (Ringle et al., 2024). The results showed that the Q^2^predict values for PE, TS, and IB were 0.194, 0.263, and 0.284, respectively, all greater than 0. These results indicate satisfactory predictive relevance for the endogenous constructs, with relatively stronger predictive performance for IB.

### 5.4. Indirect-Effect Analysis

To examine the specific indirect effects, this study used the bootstrap method. The results are presented in Table 8. The findings indicate that significant indirect associations between the antecedent variables and IB were observed through both PE and TS.

Specifically, for the indirect paths through PE, the indirect association between PS and IB through PE was significant (β = 0.064, *t* = 4.924, *p* < 0.001), as was the indirect association between HBSPT and IB through PE (β = 0.059, *t* = 4.808, *p* < 0.001). The indirect association between CHT and IB through PE was also significant (β = 0.037, *t* = 2.975, *p* < 0.01).

For the indirect paths through TS, the indirect association between PS and IB through TS was significant (β = 0.094, *t* = 6.129, *p* < 0.001), as was the indirect association between HBSPT and IB through TS (β = 0.072, *t* = 5.380, *p* < 0.001). The indirect association between CHT and IB through TS was likewise significant (β = 0.043, *t* = 3.158, *p* < 0.01).

Overall, all six specific indirect effects reached statistical significance, indicating statistically significant indirect associations between the antecedent variables and IB through both PE and TS. In terms of effect size, the PS→TS→IB path showed the largest indirect effect (β = 0.094), followed by HBSPT→TS→IB (β = 0.072) and PS→PE→IB (β = 0.064). These results should be interpreted as statistical indirect associations within the proposed model, because the cross-sectional design does not establish temporal ordering or causal mediation.

### 5.5. Moderating Effect Analysis

To examine the moderating role of SC in the relationship between PE and IB, this study further included the interaction term SC × PE in the structural model and tested its significance using the bootstrap method. The results, as shown in the direct effects Table 6, indicated that the interaction path coefficient for SC × PE→IB was not statistically significant (β = −0.053, *t* = 1.629, *p* = 0.103). This suggests that self-control did not significantly moderate the relationship between pleasure emotion and impulsive buying; therefore, the corresponding hypothesis was not supported.

### 5.6. Gender-Based Multi-Group Analysis

To examine the stability of the model across gender groups, this study conducted a multi-group analysis based on gender. The MICOM procedure was used to assess measurement invariance. As shown in Table 9, configural invariance was established for all constructs in Step 1, and the original correlation coefficients in Step 2 were all above the 5% critical values. This indicates that the model satisfied the requirements for partial measurement invariance, allowing further multi-group comparisons. In Step 3, full measurement invariance was established for IB, PE, SC, and TS, whereas CHT, PS, and HBSPT did not fully satisfy the equal mean and/or equal variance requirements.

Based on this, bootstrap MGA was further used to compare the direct paths between the male and female groups. As shown in Table 10, none of the between-group differences in the direct paths reached statistical significance (*p* > 0.05), indicating no significant gender differences were observed in the direct relationships within the model.

In addition, the between-group comparison of total indirect effects (Table 11) also showed that the differences for CHT→IB, PS→IB, and HBSPT→IB were not statistically significant (*p* > 0.05). This suggests that no significant gender differences were identified at the level of overall indirect effects.

Overall, the gender-based multi-group analysis indicates that the proposed model remained generally stable across male and female respondents. Neither the structural paths nor the total indirect effects differed significantly between male and female respondents

## 6. Discussion

### 6.1. Discussion of Findings

Based on the SOR framework and Dual-System Theory, this study examined the structural associations among consumer-perceived marketing-related cues and evaluations, internal psychological states, and impulsive buying in the context of sports livestreaming e-commerce (Evans & Stanovich, 2013; Frankish, 2010; H. Liu et al., 2025; Mehrabian & Russell, 1974). The proposed hypotheses were tested using PLS-SEM (Hair et al., 2019). The results show that consumer herding tendency, perceived scarcity, and hedonic benefits of sales promotion tools were positively associated with both pleasure emotion and trust in seller (C.-H. Lee & Chen, 2021; Lin et al., 2022; Xia et al., 2024). Pleasure emotion and trust in seller were further positively associated with impulsive buying, whereas self-control was negatively associated with impulsive buying. These findings suggest that impulsive buying among consumers with sports livestreaming shopping experience is related to both positive affective experience and trust-based evaluation, while self-control appears to be more closely related to impulsive buying as a direct negative correlate (Baumeister, 2002; S. Chen et al., 2022; Flight et al., 2012; Huang et al., 2024; C. Wang et al., 2025a; Yang et al., 2025).

The indirect-effect analysis showed that consumer herding tendency, perceived scarcity, and hedonic benefits of sales promotion tools were indirectly associated with impulsive buying through both pleasure emotion and trust in seller. Notably, the indirect associations through trust in seller were generally stronger than those through pleasure emotion. This pattern suggests that impulsive buying among consumers with sports livestreaming shopping experience is associated not only with pleasure-related affective experience but also with consumers’ trust-based evaluation of sellers (C.-H. Lee & Chen, 2021; Xia et al., 2024). From an SOR perspective, these results can be organized as associations among consumer-perceived cue-related conditions, psychological states, and behavioral responses. From a Dual-System Theory perspective, the findings suggest that impulsive buying may be related to both affective responses and trust-related evaluative processes. However, because this study used a cross-sectional self-report design, these findings should be interpreted as indirect associations rather than evidence of causal mediation.

Contrary to expectations, the moderating effect of self-control was not supported. Although self-control was directly and negatively associated with impulsive buying (Tangney et al., 2018), it did not significantly moderate the relationship between pleasure emotion and impulsive buying. One possible explanation is that self-control may be more appropriately interpreted as a general negative correlate of impulsive buying, rather than as a boundary condition that weakens the association between pleasure emotion and impulsive buying (Baumeister, 2002). In sports livestreaming shopping, real-time interaction, collective participation, time-limited promotions, and sport-related excitement may create salient situational experiences (Lin et al., 2022; H. Liu et al., 2025; Xia et al., 2024). These experiences may be associated with a relatively stable link between pleasure-related affect and impulsive buying across different levels of self-control. Thus, self-control appears to be more relevant as a direct negative correlate of impulsive buying than as a moderator of the pleasure emotion–impulsive buying relationship. In addition, the gender-based multi-group analysis revealed no significant differences between male and female consumers, suggesting that the examined associations were broadly similar across gender groups in this sample.

Overall, the findings can be interpreted within the SOR framework by organizing the observed associations among consumer-perceived marketing-related cues and evaluations, psychological states, and impulsive buying (Van Tran et al., 2023; Xia et al., 2024). Dual-System Theory also provides a useful interpretive lens for understanding why impulsive buying among consumers with sports livestreaming shopping experience is associated with both pleasure-related affect and trust-based evaluation (Evans & Stanovich, 2013). Taken together, these findings suggest that impulsive buying among consumers with sports livestreaming shopping experience reflects the joint relevance of affective experience and trust-based evaluation.

### 6.2. Theoretical Implications

This study offers three main theoretical implications. First, by applying the SOR framework to consumers with sports livestreaming shopping experience, this study adds to existing SOR research by suggesting that consumer-perceived marketing-related cues and evaluations are associated with impulsive buying through internal psychological states. The findings indicate that pleasure emotion and trust in seller may help explain the association between marketing-related perceptions and impulsive buying (C.-H. Lee & Chen, 2021; Mehrabian & Russell, 1974).

Second, by integrating Dual-System Theory, this study offers a theoretical rationale for considering both affective and evaluative psychological processes in sports livestreaming e-commerce. Specifically, the findings suggest that impulsive buying is associated not only with pleasure-related affect but also with trust-based evaluation. This implication helps move the discussion beyond a single affective explanation of impulsive buying and highlights the relevance of trust in understanding consumers’ purchase-related responses in livestreaming environments (Evans & Stanovich, 2013; Wongkitrungrueng & Assarut, 2020).

Third, the stronger indirect associations through trust in seller suggest that trust-based evaluation may play a particularly relevant role in understanding impulsive buying in sports livestreaming e-commerce. In livestreaming shopping, consumers often rely on sellers, streamers, and real-time information when evaluating products and transactions. Therefore, trust in seller may be closely related to consumers’ willingness to make impulsive purchases. In contrast, the non-significant moderating effect of self-control and the non-significant gender-based multi-group differences suggest limited evidence for these boundary conditions in the present sample. These findings indicate that future research should identify additional contextual or individual-level moderators, such as fandom, product involvement, streamer credibility, or platform characteristics, to better explain when and for whom impulsive buying is more likely to occur.

### 6.3. Practical Implications

The findings offer practical implications for responsible sports livestreaming e-commerce. First, platforms and sellers should manage popularity cues, scarcity information, and promotional claims transparently. Popularity indicators, stock information, countdowns, and promotional benefits should be truthful, clearly disclosed, and updated in real time. Such practices may help maintain consumer trust and support long-term seller–consumer relationships.

Second, the stronger association involving trust in seller suggests that impulsive buying in sports livestreaming e-commerce is related not only to pleasure emotion but also to consumers’ evaluation of seller credibility. Therefore, sellers should strengthen trust by providing accurate product information, professional explanations, authentic demonstrations, clear transaction rules, and reliable after-sales service.

Finally, the negative association between self-control and impulsive buying suggests the value of consumer-supportive platform design. Platforms may provide clear product comparisons, transparent price information, purchase confirmation prompts, spending reminders, and accessible refund or complaint channels. These measures can support more informed purchasing decisions while preserving the interactive advantages of sports livestreaming e-commerce.

## 7. Conclusions

This study examined the psychological processes associated with consumers’ impulsive buying in sports livestreaming e-commerce. The results show that consumer herding tendency, perceived scarcity, and hedonic benefits of sales promotion tools were positively associated with both pleasure emotion and trust in seller. Pleasure emotion and trust in seller were positively associated with impulsive buying, whereas self-control was negatively associated with impulsive buying. The indirect-effect analysis further showed that these consumer-perceived marketing-related cues and evaluations were indirectly associated with impulsive buying through pleasure emotion and trust in seller, with the indirect associations through trust in seller being generally stronger. However, the moderating effect of self-control was not supported, and the gender-based multi-group analysis revealed no significant differences.

Overall, this study suggests that impulsive buying among consumers with sports livestreaming shopping experience is associated with both pleasure-related affect and trust-based evaluation. The findings can be interpreted through the SOR framework and Dual-System Theory, while highlighting the relevance of trust in seller for understanding consumers’ impulsive buying tendencies in this context.

### Research Limitations and Future Directions

This study has several limitations. First, the sample consisted of university students from eastern China, which may limit the generalizability of the findings. Although this group is relevant to sports livestreaming e-commerce, their consumption patterns may differ from those of older consumers, working adults, higher-income consumers, or consumers from other regions and cultural contexts. Future research should test the proposed model using more diverse samples.

Second, although respondents were asked to recall their most recent sports livestreaming shopping experience, the survey did not collect detailed contextual information, such as product categories, platform types, streamer or seller characteristics, live-event versus sports-product livestreaming formats, or fan identification with particular sports, athletes, teams, or brands. Therefore, the sport-specific interpretation of the findings should be made cautiously, as the present study did not directly compare sports livestreaming e-commerce with other livestreaming shopping contexts. Accordingly, sport-specific features such as fandom, team identification, athlete or brand endorsement, event-based promotions, limited-edition products, sport-product involvement, seller type, and perceived authenticity concerns provide useful contextual background for interpreting the findings, but their explanatory role should be directly tested in future research. Future research should compare sports and non-sport livestreaming contexts and directly examine these sport-specific factors.

Third, this study used cross-sectional same-source self-report data, which limits its ability to examine temporal ordering, causal relationships, and dynamic changes among the variables. In addition, because all substantive variables were collected from the same respondents using the same questionnaire, common method variance cannot be fully ruled out. Although Harman’s single-factor test and VIF diagnostics were conducted as preliminary checks, the questionnaire did not include a theoretically unrelated marker variable, and an unmeasured latent method factor was not estimated. Therefore, the indirect-effect results should be interpreted as statistical indirect associations rather than evidence of temporal or causal mediation. Future studies should adopt longitudinal, experimental, multi-source, or behavioral-data designs and apply more rigorous procedural and statistical controls for common method variance.

Fourth, the conceptual proximity among several self-reported constructs should be acknowledged. Although the measurement model showed acceptable reliability, convergent validity, discriminant validity, and HTMT results, these statistical results do not fully eliminate the possibility that some constructs share related affective or evaluative content. For example, some items related to hedonic sales promotion and pleasure emotion may involve closely related affective or evaluative elements. Future research should develop more precise measures to better distinguish consumer-perceived marketing-related cues, affective experiences, and evaluative psychological states.

Finally, the non-significant moderating effect of self-control and the non-significant gender-based multi-group differences suggest that future research should examine additional boundary conditions. Potential moderators may include product involvement, fan identification, platform type, perceived risk, seller credibility, promotion transparency, persuasion knowledge, cultural background, or sports involvement. These factors may help clarify when and for whom impulsive buying is more likely to occur in sports livestreaming e-commerce. Taken together with the limitations noted above, these non-significant boundary-condition findings suggest that the explanatory scope of the current model should be interpreted cautiously.

## Figures and Tables

**Table 1:** Measurement items and sources of constructs.

Construct	Item	Scales	Source
Consumer Herding Tendency	CHT1	I tend to follow other consumers’ purchasing decisions in sports livestreaming e-commerce environments.	(X. Chen et al., 2025; Duong et al., 2025)
	CHT2	If many consumers buy a product, I am more likely to buy it as well.	
	CHT3	Knowing that many others have purchased a product increases my likelihood of choosing it.	
	CHT4	I feel more confident choosing a product when most consumers choose it.	
Perceived scarcity	PS1	I think that the current supply of this product is small.	(Wu et al., 2012)
	PS2	I think that this product is selling out soon.	
	PS3	I think that many people will buy this product.	
	PS4	I feel that the limited edition of this product will cause many people to buy.	
Hedonic benefits of sales promotion tools	HBSPT1	I feel proud to purchase product X with Sale promotion.	(Sinha & Verma, 2020)
	HBSPT2	I like trying new brands of product X on the availability of Sale promotion.	
	HBSPT3	I enjoy purchasing product X with Sale promotion.	
	HBSPT4	I feel pleased to purchase product X with Sale promotion.	
Trust in seller	TS1	I believe in the information that the seller provides through sports livestreaming e-commerce.	(Sullivan & Kim, 2018)
	TS2	I can trust sellers who use sports livestreaming e-commerce.	
	TS3	I believe that sellers who use sports livestreaming e-commerce are trustworthy.	
	TS4	I do not think that sellers who use sports livestreaming e-commerce would take advantage of me.	
Pleasure Emotion	PE1	The sports livestreaming e-commerce made me feel the joy of the shopping process.	(L. Li et al., 2024)
	PE2	I felt exhilarated when the anchor promoted products and everyone made purchases together.	
	PE3	The dynamic and engaging content of sports live streaming e-commerce made it intriguing for me.	
	PE4	Watching sports live streaming makes time fly by, and I forget all my worries.	
Impulsive Buying	IB1	I often find myself succumbing to impulse buying tendencies when browsing through products on this livestreaming channel.	(L. Li et al., 2024)
	IB2	During the aforementioned live streaming, I made an impulse purchase without taking the time to fully consider and evaluate the product.	
	IB3	There were certain products that had never caught my attention before; however, upon seeing the anchor’s recommendations, I found myself buying them impulsively.	
	IB4	Following the anchor’s informative and eloquent presentation, I was struck with a sudden realization of my need for a particular product, which compelled me to make a purchase.	
Self-control	SC1 (R)	I do things that bring me happiness but are harmful to me (R)	(S. Chen et al., 2022)
	SC2 (R)	Sometimes I get distracted by having fun and cannot finish the task on time (R)	
	SC3 (R)	Sometimes I cannot help but do things that I know are wrong (R)	
	SC4 (R)	I often act without thinking (R)	

Note: R indicates reverse-coded items.

**Table 2:** Respondents’ demographic information.

Category	Subcategory	N	Percentage (%)
Gender	Male	326	42.1
	Female	449	57.9
Age	Under 18	76	9.8
	19–22	511	65.9
	23–26	60	7.7
	27–30	89	11.5
	31 and above	39	5
Education Level	Junior College	134	17.3
	Bachelor’s Degree	480	61.9
	Master’s Degree	120	15.5
	Doctoral Degree	41	5.3
Average Monthly Living Expenses	Below RMB 3000	559	72.1
	RMB 3000–5999	50	6.5
	RMB 6000–8999	47	6.1
	RMB 9000–11,999	53	6.8
	RMB 12,000 and above	66	8.5
Weekly Viewing Frequency of sports livestreaming e-commerce	1–2 times	648	83.6
	3–4 times	75	9.7
	5–6 times	15	1.9
	7 times and above	37	4.8
Monthly Spending on Live-Streaming E-commerce	RMB 500 or below	562	72.5
	RMB 501–1500	128	16.5
	RMB 1501–3000	49	6.3
	RMB 3001–5000	20	2.6
	Above RMB 5000	16	2.1

**Table 3:** Results of measurement model analysis.

	Cronbach’s Alpha	Composite Reliability (rho_a)	Composite Reliability (rho_c)	Average Variance Extracted (AVE)	Item	Outer Loadings	VIF
Consumer Herding Tendency (CHT)	0.810	0.813	0.875	0.636	CHT1	0.809	1.716
					CHT2	0.757	1.544
					CHT3	0.812	1.662
					CHT4	0.811	1.717
Impulsive Buying (IB)	0.809	0.809	0.875	0.636	IB1	0.792	1.609
					IB2	0.776	1.578
					IB3	0.841	1.995
					IB4	0.779	1.667
Pleasure Emotion (PE)	0.840	0.841	0.893	0.676	PE1	0.827	1.988
					PE2	0.815	1.860
					PE3	0.816	1.939
					PE4	0.830	2.001
Perceived scarcity (PS)	0.845	0.845	0.896	0.683	PS1	0.805	1.808
					PS2	0.801	1.969
					PS3	0.861	2.379
					PS4	0.836	1.990
Self-control (SC)	0.899	0.904	0.929	0.767	SC1(R)	0.869	2.401
					SC2(R)	0.901	2.835
					SC3(R)	0.876	2.626
					SC4(R)	0.857	2.404
Hedonic benefits of sales promotion tools (HBSPT)	0.830	0.831	0.887	0.663	HBSPT1	0.810	1.791
					HBSPT2	0.818	1.750
					HBSPT3	0.802	1.703
					HBSPT4	0.825	1.854
Trust in seller (TS)	0.856	0.856	0.903	0.699	TS1	0.860	2.219
					TS2	0.799	1.698
					TS3	0.837	1.987
					TS4	0.846	2.091

**Table 4:** Fornell–Larcker Criterion.

	CHT	IB	PE	PS	SC	HBSPT	TS
CHT	0.798						
IB	0.438	0.797					
PE	0.304	0.420	0.822				
PS	0.399	0.433	0.370	0.826			
SC	−0.285	−0.307	−0.237	−0.287	0.876		
HBSPT	0.347	0.469	0.352	0.384	−0.256	0.814	
TS	0.337	0.453	0.357	0.444	−0.280	0.392	0.836

**Table 5:** HTMT.

	CHT	IB	PE	PS	SC	HBSPT	TS	SC × PE
CHT								
IB	0.540							
PE	0.367	0.508						
PS	0.484	0.524	0.438					
SC	0.332	0.358	0.269	0.327				
HBSPT	0.423	0.571	0.420	0.458	0.294			
TS	0.404	0.544	0.421	0.522	0.317	0.465		
SC × PE	0.031	0.037	0.067	0.089	0.020	0.050	0.038	

**Table 6:** Path Coefficients of Direct Effects.

	Original Sample (O)	Sample Mean (M)	Standard Deviation (STDEV)	T Statistics (|O/STDEV|)	*p* Values
CHT→PE	0.136	0.137	0.039	3.489	0.000
CHT→TS	0.138	0.138	0.038	3.644	0.000
PE→IB	0.275	0.275	0.033	8.297	0.000
PS→PE	0.233	0.234	0.038	6.166	0.000
PS→TS	0.301	0.302	0.034	8.812	0.000
SC→IB	−0.154	−0.156	0.032	4.819	0.000
HBSPT→PE	0.215	0.216	0.036	5.966	0.000
HBSPT→TS	0.229	0.230	0.033	6.976	0.000
TS→IB	0.314	0.315	0.035	9.084	0.000
SC × PE→IB	−0.053	−0.053	0.033	1.629	0.103

**Table 7:** Model Explanatory Power and Predictive Relevance Indicators.

	R^2^	R^2^ Adjusted	Q^2^ Predict	Predictor→Outcome	f^2^
IB	0.306	0.303	0.284	SC→IB	0.031
				PE→IB	0.092
				TS→IB	0.118
				SC × PE→IB	0.004
PE	0.204	0.200	0.194	PS→PE	0.053
				HBSPT→PE	0.047
				CHT→PE	0.019
TS	0.270	0.267	0.263	PS→TS	0.096
				HBSPT→TS	0.058
				CHT→TS	0.021

**Table 8:** Bootstrap Results for Specific Indirect Effects.

	Original Sample (O)	Sample Mean (M)	Standard Deviation (STDEV)	T Statistics (|O/STDEV|)	*p* Values
PS→PE→IB	0.064	0.064	0.013	4.924	0.000
HBSPT→PE→IB	0.059	0.059	0.012	4.808	0.000
CHT→TS→IB	0.043	0.044	0.014	3.158	0.002
PS→TS→IB	0.094	0.095	0.015	6.129	0.000
CHT→PE→IB	0.037	0.038	0.013	2.975	0.003
HBSPT→TS→IB	0.072	0.072	0.013	5.380	0.000

**Table 9:** Results of Invariance Measurement Testing Using Permutation.

Constructs	Configurational Invariance (Step 1)	Compositional Invariance (Step 2)		Partial Measurement Invariance	Equal Mean Assessment (Step 3a)		Equal Variance Assessment (Step 3b)		Full Measurement Invariance
		Original Correlation	5.00%		Original Differences	Confidence Interval	Original Differences	Confidence Interval	
CHT	YES	1.000	0.998	YES	−0.170	[−0.122; 0.126]	0.166	[−0.154; 0.157]	NO/NO
IB	YES	1.000	0.999	YES	−0.013	[−0.123; 0.123]	0.044	[−0.137; 0.134]	YES/YES
PE	YES	1.000	0.999	YES	−0.027	[−0.116; 0.110]	−0.051	[−0.153; 0.133]	YES/YES
PS	YES	1.000	0.999	YES	−0.154	[−0.113; 0.117]	0.177	[−0.148; 0.161]	NO/NO
SC	YES	1.000	0.999	YES	−0.027	[−0.130; 0.113]	−0.138	[−0.168; 0.159]	YES/YES
HBSPT	YES	0.999	0.999	YES	−0.198	[−0.121; 0.113]	−0.005	[−0.161; 0.155]	NO/YES
TS	YES	1.000	1.000	YES	−0.103	[−0.123; 0.114]	−0.024	[−0.139; 0.135]	YES/YES

Note: Step 1: Normally, this is automatically established. Step 2: The original correlation is higher than 5% and the permutation *p*-value is higher than 0.05. Step 3: (a) Not all confidence intervals of latent variable score means include the original differences value, so there are not equal means. (b) Not all confidence intervals of latent variable score variances include the original differences value, so there are not equal variances.

**Table 10:** Bootstrap MGA Results for Direct Paths.

	Difference (Group_1 Gender Male—Group_2 Gender Female)	1-Tailed (Group_1 Gender Male vs. Group_2 Gender Female) *p* Value	2-Tailed (Group_1 Gender male vs. Group_2 Gender Female) *p* Value
CHT→PE	0.038	0.313	0.625
CHT→TS	0.018	0.406	0.813
PE→IB	−0.106	0.943	0.114
PS→PE	0.108	0.078	0.155
PS→TS	−0.002	0.509	0.982
SC→IB	−0.047	0.762	0.475
SC × PE→IB	−0.005	0.528	0.943
HBSPT→PE	−0.009	0.549	0.901
HBSPT→TS	0.004	0.475	0.950
TS→IB	−0.034	0.683	0.635

**Table 11:** Bootstrap MGA Results for Total Indirect Effects.

	Difference (Group_1 Gender Male—Group_2 Gender Female)	1-Tailed (Group_1 Gender Male vs. Group_2 Gender Female) *p* Value	2-Tailed (Group_1 Gender Male vs. Group_2 Gender Female) *p* Value
CHT→IB	−0.004	0.545	0.911
PS→IB	−0.008	0.584	0.831
HBSPT→IB	−0.032	0.825	0.35

## Data Availability

The data presented in this study are available on reasonable request from the corresponding author. The data are not publicly available due to privacy and ethical restrictions.
